# In silico analysis for factors affecting anti-malarial penetration into red blood cells

**DOI:** 10.1186/s12936-020-03280-y

**Published:** 2020-06-23

**Authors:** Natapol Pornputtapong, Bovornpat Suriyapakorn, Anchisa Satayamapakorn, Kanidsorn Larpadisorn, Pariyachut Janviriyakul, Phisit Khemawoot

**Affiliations:** 1 Department of Biochemistry and Microbiology, Faculty of Pharmaceutical Sciences, Chulalongkorn University, Bangkok, Thailand; 2 Vaccine and Therapeutic Protein, The Special Task Force for Activating Research, Faculty of Pharmaceutical Sciences, Chulalongkorn University, Bangkok, Thailand; 3 Center of Excellence in Systems Biology, Faculty of Medicine, Chulalongkorn University, Bangkok, Thailand; 4 Department of Pharmacy Practice, Faculty of Pharmaceutical Sciences, Chulalongkorn University, Bangkok, Thailand; 5 Preclinical Pharmacokinetics and Interspecies Scaling for Drug Development Research Unit, Chulalongkorn University, Bangkok, Thailand; 6 Chakri Naruebodindra Medical Institute, Faculty of Medicine Ramathibodhi Hospital, Mahidol University, Bang Phli, Samut Prakarn, 10540 Thailand

**Keywords:** Antimalarials, Red blood cell, Penetration

## Abstract

**Background:**

Malaria is a parasitic disease that produces significant infection in red blood cells. The objective of this study is to investigate the relationships between factors affecting the penetration of currently available anti-malarials into red blood cells.

**Methods:**

Fifteen anti-malarial drugs listed in the third edition of the World Health Organization malaria treatment guidelines were enrolled in the study. Relationship analysis began with the prioritization of the physicochemical properties of the anti-malarials to create a multivariate linear regression model that correlates the red blood cell penetration.

**Results:**

It was found that protein binding was significantly correlated with red blood cell penetration, with a negative coefficient. The next step was repeated analysis to find molecular descriptors that influence protein binding. The coefficients of the number of rotating bonds and the number of aliphatic hydrocarbons are negative, as opposed to the positive coefficients of the number of hydrogen bonds and the number of aromatic hydrocarbons. The p-value was less than 0.05.

**Conclusions:**

Anti-malarials with a small number of hydrogen bonds and aromatic hydrocarbons, together with a high number of rotatable bonds and aliphatic hydrocarbons, may have a higher tendency to penetrate the red blood cells.

## Background

Malaria is an infectious disease generated by *Plasmodium* spp., which continues to be a public health problem in Thailand. The 2018 Thai guidelines for the treatment of malaria recommend artemisinin-based combination therapy as the first-line regimen [1]. Currently, the first-generation artemisinin derivatives, including artemisinin, artemether, arteether, artesunate, and dihydroartemisinin, are still widely used [2]. Each derivative penetrates the red blood cells differently and has a distinctive ability to kill malaria parasites [3, 4]. This study aims to determine the factors that confer a different capability to enter the red blood cells. Therefore, we selected 15 anti-malarial drugs according to the World Health Organization (WHO) malaria treatment guidelines for this study [5]. These include artemisinin, dihydroartemisinin, artemether, arteether, artesunate, chloroquine, mefloquine, primaquine, amodiaquine, piperaquine, quinine, sulfadoxine, pyrimethamine, doxycycline, and proguanil. The screening procedures here identified the three most influential physicochemical parameters that could affect erythrocyte penetration. Information obtained from this study would be beneficial for the development of new anti-malarial drugs that are more effective in penetrating red blood cells.

## Methods

### Data collection

The WHO’s malaria treatment guidelines recommend 15 anti-malarial drugs for first-line malaria treatment. Structures and molecular weights were mostly retrieved from the PubChem database [6, 7]. The structures were downloaded in InChI and SMILES formats, which were more convenient for molecular descriptor calculation. Protein binding, water solubility, and red blood cell to plasma drug concentration ratio were gathered from various sources as shown in Table 1. These parameters were converted into numeric data for statistical analysis. The charge state properties of drugs and the acid–base characteristics were not explicitly described in the learning model, but implicated in the hypothesis as LogP and protein binding, which were included in the model and subject to the charge and acid–base characteristics of drugs. There is also a possibility of active drug transporters, but the information is sparse and limited; therefore, this feature was not included in the model.

**Table 1:** Physicochemical parameters of anti-malarial drugs

Drug	Molecular weight (g/mol)	RBC ratio	LogP [8]	Protein binding (%)	Water solubility (mg/L)
Amodiaquine	355.8 [6]	1.313 [7]	5.1792	92 [9]	2.83 [6]
Arteether	312.41 [6]	0.23 [10]	3.2309	78.7 [11]	1700 [12]
Artemether	298.38 [6]	0.28 [10]	2.8408	95.4 [6]	12.1 [6]
Artemisinin	282.34 [6]	0.49 [10]	2.3949	82.5 [13]	51.9 [14]
Artesunate	384.18 [6]	0.71 [10]	2.6024	88 [15]	56.2 [6]
Chloroquine	319.88 [6]	4.8 [16]	4.8106	52.5 [6]	0.14 [6]
Dihydroartemisinin	284.35 [6]	0.52 [10]	2.1867	82 [17]	3160 [18]
Doxycycline	444.44 [6]	0.37 [19]	0.5458	90 [20]	630 [6]
Mefloquine	378.32 [6]	2.8 [21]	4.4479	98 [6]	6.212 [6]
Piperaquine	535.52 [6]	1.5 [22]	5.4241	97 [23]	0 [24]
Primaquine	259.35 [6]]	1 [25]	2.7827	75 [26]	1300 [6]
Proguanil	253.73 [20]	4.9 [27]	2.209	75 [20]	156 [20]
Pyrimethamine	248.71 [6]	0.42 [28]	2.3542	87 [29]	121 [6]
Quinine	324.42 [20]	1.89 [30]	3.1732	70 [20]	334 [20]
Sulfadoxine	310.33 [6]	0.163 [31]	0.8768	90 [32]	296 [20]

RBC, red blood cell

### Molecular descriptor calculation

Chemical structure, which is a graphical notation of the compound, is complicated to use in mathematical calculations. To make computation feasible, a molecular descriptor is created. A molecular descriptor is a numerical notation associated with the chemical constitution; it is used in machine learning for correlation calculations for a compound regarding physical properties and biological activities. LogP, Number of rotatable bonds (Rot), number of hydrogen bond acceptors (HBA), number of hydrogen bond donors (HBD), number of aliphatic carbocycles (AliCarbo), number of aliphatic heterocycles (AliHet), number of aromatic carbocycles (AroCarbo), number of aromatic heterocycles (AroHet), and number of saturated carbocycles (SatCarbo) were calculated using a python package RDKit [8] as shown in Table 2.

**Table 2:** Molecular descriptors of anti-malarial drugs

Drug	Rot	HBA	HBD	Ali carbo	Ali het	Aro carbo	Aro het	Sat carbo
Amodiaquine	6	4	2	0	0	2	1	0
Arteether	2	5	0	1	4	0	0	1
Artemether	1	5	0	1	4	0	0	1
Artemisinin	0	5	0	1	4	0	0	1
Artesunate	4	7	1	1	4	0	0	1
Chloroquine	8	3	1	0	0	1	1	0
Dihydroartemisinin	0	5	1	1	4	0	0	1
Doxycycline	2	9	7	3	0	1	0	1
Mefloquine	2	3	2	0	1	1	1	0
Piperaquine	6	6	0	0	2	2	2	0
Primaquine	6	4	2	0	0	1	1	0
Proguanil	2	2	5	0	0	1	0	0
Pyrimethamine	2	3	3	0	0	1	1	0
Quinine	4	4	1	0	3	1	1	0
Sulfadoxine	5	7	2	0	0	1	1	0

Rot, number of rotatable bonds; HBA, number of hydrogen bond acceptors; HBD, number of hydrogen bond donors; AliCarbo, number of aliphatic carbocycles; AliHet, number of aliphatic heterocycles; AroCarbo, number of aromatic carbocycles; AroHet, number of aromatic heterocycles; and SatCarbo, number of saturated carbocycles

### Data analysis

Several independent variables, including physicochemical properties and chemical descriptors, were retrieved. Using all variables to fit the model might lead to overfitting. Variables were selected based on relative importance, which was calculated using the relaimpo package in R programming language [33] for each independent variable. The relative importance is a comparative score among independent variables themselves to rank the effect of changing the variables to the right prediction.

An extreme gradient boosting tree regression, a non-linear regression method from Extreme Gradient Boosting (XGBoost) library [34], was used to describe the relationship a drug to its red blood cell to plasma drug concentration ratio. A non-linear regression method was chosen to challenge with a non-linear property of pharmacokinetic distribution processes, which might be a part of the relationship between physicochemical properties of the drug and red blood cell distribution. The first model was computed using relative-importance-selected physicochemical properties as independent variables and red blood cells to plasma drug concentration ratio as the dependent variable. The model was optimized using a black-box optimization method implemented in the Optuna framework [35] objected to mean squared error (MSE). The optimized model was evaluated using a permutation test [36] with five k-fold for 1000 rounds before fitting. The fitted extreme gradient boosting tree regression model was further analysed using a SHapley Additive exPlanations (SHAP) algorithm [37] for unveiling relationship of each independent variable to the dependent variable.

A linear regression model was used to describe the relationship of molecular descriptors of anti-malarial drugs to its protein binding. Before fitting the model, a boxplot and a correlation plot were constructed, and independent variables were tested for normality using the Shapiro–Wilks test. The model was fitted into multiple linear regression models, as shown in Eq. 1 by lm function. The statistical analyses were performed using R version 3.4.0 [38]. The second model was trained using relative-importance-selected molecular descriptors of anti-malarial drugs as independent variables and protein binding as dependent variables to elucidate the structural features and relationships among them. Multiple R-squared, adjusted R-squared, and F-statistics were calculated by lm function after the model was fitted.

1 $$Y = \beta_{0} + \mathop \sum \limits_{i = 1}^{n} \beta_{i} x_{i} + \varepsilon$$

Equation 1, The multiple linear regression model. Y represents an independent variable. β_0_ represents an intercept. β_i_ represents a regression coefficient. ε represents error.

## Results

### Study on the relationships between factors influencing the red blood cell penetration of anti-malarial drugs

**Fig. 1:**
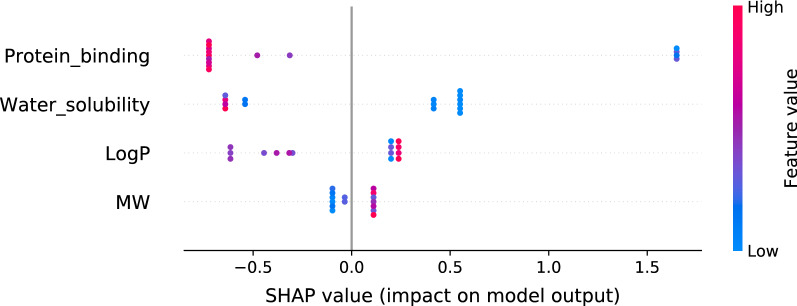
SHAP value presenting the impact of each feature to drug red blood cell/plasma ratio The feature on the top is the highest important feature for the prediction, and spot color is represent feature value. The positive SHAP value shows the impact of the feature on increasing of the dependent variable in the prediction

From the important factors analysis, protein binding, logP, water solubility, and molecular weight were considered as the most important factors and used as a feature set for extreme gradient boosting model construction. The model was optimized, then the permutation test with five-fold validation was performed. The average MSE and R-squared were 1.90 and 0.27, with p-0.037 and 0.009, respectively, showed a statistically significant model. From a summary plot from the SHAP algorithm, protein binding was shown to be the most important factor for red blood cell penetration properties prediction. The higher value of protein binding impacts lowering the drug red blood cell/plasma ratio, as shown in Fig. 1.

### Study of the relationships between the molecular descriptors affecting protein binding

From the important factors analysis, the number of rotatable bonds was the most important, followed by the number of hydrogen bond acceptors. Then, testing for the cross-validation and statistical significance of the correlation coefficients showed that the number of rotatable bonds, hydrogen bond acceptors, aliphatic hydrocarbons, and aromatic hydrocarbons were significant factors correlated with protein binding as shown in Table 3.

**Table 3:** Multiple linear regression of anti-malarial drugs and their abilities in protein binding

Parameters	Estimate	SE	t-value	p-value
(Intercept)	0.599	0.098	6.123	0.000174 *
Rotatable bond	− 0.047	0.014	− 3.381	0.00811 *
Hydrogen bond acceptor	0.052	0.020	2.584	0.029 *
Aliphatic hydrocarbon	− 0.148	0.063	− 2.337	0.044 *
Aromatic hydrocarbon	0.183	0.072	2.517	0.033 *
Saturated hydrocarbon	0.185	0.142	1.297	0.227

Protein binding = − 0.047 × Rotatable bond + 0.052 × Hydrogen bond acceptor–0.148 × Aliphatic hydrocarbon + 0.183 × Aromatic hydrocarbon + 0.185 × Saturated hydrocarbon + 0.599

Residual standard error: 0.083 on 9 degrees of freedom

Multiple R-squared: 0.692, Adjusted R-squared: 0.521

F-statistic: 4.05 on 5 and 9 DF, p-value: 0.033 *

* Significance value at 0.05

## Discussion

Machine learning is a powerful approach that widely used in many fields in the sciences for finding valuable information from data. The aims of a machine learning model development can be both to build a robust predictive model and to explain a relationship of features to outcomes. To create a predictive model needs a vast dataset to be learned by the model. While the anti-malarial drug is orphan, so the data of the drug is limited. Thus, the objective of this analysis was to investigate features that could involve drug-red blood cell partition, not to build a robust predictive model due to a limitation of data.

From the extreme gradient boosting regression of anti-malarial drugs and their abilities in red blood cell penetration, the R-squared was 0.27. Also, the multiple linear regression of anti-malarial drugs, and their abilities in protein binding Adjusted R-squared was 0.521. These could illustrate that the predictive power of the model is incompetent. However, we can find the essential feature protein binding and some statistically significant chemical descriptors from the model, which demonstrate the relationship of them to the drug-red blood cell partition. This conclusion might lead to new potential substances that can protect against malaria in the future.

According to the analysis of factors affecting penetration of 15 anti-malarial drugs into red blood cells, we found that protein binding dominantly affects the penetration. Low protein binding causes an increased level of free drug in plasma, allowing the drug to distribute and penetrate into red blood cells. This finding is consistent with the hypotheses in previous studies of different drugs. A study of cyclosporin A revealed that the level of free drug was directly related to the concentration of the drug in red blood cells, in similar manner to another study of phenytoin [39–41]. Moreover, analysis of molecular descriptors affecting protein-binding property showed that the number of rotatable bonds, hydrogen bond acceptors, aliphatic hydrocarbons and aromatic hydrocarbons was significantly related to the protein-binding property of the drug. This property decreased with a lack of hydrogen bond acceptors and aromatic hydrocarbons; on the other hand, it increased with a lack of rotatable bonds and aliphatic hydrocarbons.

Approximately 50% of the protein in plasma is albumin. This protein plays an important role in binding to unbound drugs in plasma. There are two major binding sites in the albumin structure. The first site tends to fit with large drug molecules, while the other one is less flexible and stereo specifically bound to the drug [41]. It is implied that the drug with large size and less flexibility has higher ability to bind to a protein. In this study, a molecule containing a higher number of rotatable bonds had less ability to bind to plasma proteins, as the molecule was flexible. The number of hydrogen bond acceptors is directly related to protein-binding property; thus, the fewer hydrogen bond acceptors, the higher the red blood cell penetration. The study of Samari et al. found that Van der Waals forces and hydrogen bonds were dominant in the binding between amodiaquine and albumin in plasma [42]. The results presented here are also consistent with a previous study which found that drugs with a low tendency to create hydrogen bonds had increased penetration into red blood cells [43]. As for the number of aliphatic and aromatic hydrocarbons, molecules with a high number of aliphatic hydrocarbons and a low number of aromatic hydrocarbons would have decreased protein-binding property, facilitating penetration into red blood cells. This concept was mentioned in a previous study; a drug containing not more than two aromatic hydrocarbons will have more unbound drug in plasma than a drug containing more than two aromatic hydrocarbons. It will also tend to bypass metabolism in the liver, leading to high concentration of the drug in plasma [44].

In terms of pharmacokinetics and pharmacodynamics, the efficacy of an antimicrobial drug generally depends on its concentration and duration of exposure. Likewise, the efficacy of artemisinin derivatives was most related to its maximum concentration in plasma [23]. More unbound drug in the plasma would be a factor that could lead to a higher concentration of the drug at the targeted site, which for an anti-malarial drug is the red blood cell. Accumulation of the drug in red blood cells increased its half-life and consequently increased the efficacy of the drug actions. In a practical aspect, anti-malarial drugs containing higher numbers of rotatable bonds and aliphatic hydrocarbons, and lower numbers of hydrogen bond acceptors and aromatic hydrocarbons, would have less protein-binding property. Therefore, more drug will penetrate through the red blood cells, facilitating its pharmacodynamic activities.

## Conclusions

The most influential physicochemical factor for the penetration of anti-malarial drugs into red blood cells is protein binding. The less a drug is bound to protein, the more it is available in free form, which can penetrate into the red blood cell. For molecular descriptors affecting protein binding, drugs with a small number of hydrogen bond acceptors and aromatic hydrocarbons, together with a high number of rotatable bonds and aliphatic hydrocarbons, may have a higher amount of free drug in the plasma available to penetrate into the red blood cell.

## Data Availability

The data that support the findings of this study are available in the manuscript.

## Abbreviations

AliCarbo: Number of aliphatic carbocycles
AliHet: Number of aliphatic heterocycles
AroCarbo: Number of aromatic carbocycles
AroHet: Number of aromatic heterocycles
HBA: Number of hydrogen bond acceptors
HBD: Number of hydrogen bond donors
InChI: IUPAC international chemical identifier
LogP: Partition coefficient
Pb: Protein binding
RBC: Red blood cells
RMSE: Root mean square error
Rot: Number of rotatable bonds
SatCarbo: Number of saturated carbocycles
SE: Standard error
SMILES: Simplified molecular input line-entry system
